# Late-Course Adaptive Adjustment Based on Metabolic Tumor Volume Changes during Radiotherapy May Reduce Radiation Toxicity in Patients with Non-Small Cell Lung Cancer

**DOI:** 10.1371/journal.pone.0170901

**Published:** 2017-01-26

**Authors:** Linlin Xiao, Ning Liu, Guifang Zhang, Hui Zhang, Song Gao, Zheng Fu, Suzhen Wang, Qingxi Yu, Jinming Yu, Shuanghu Yuan

**Affiliations:** 1 School of Medicine and Life Sciences, University of Jinan-Shandong Academy of Medical Sciences, Jinan, Shandong, China; 2 Department of Radiation Oncology, Shandong Cancer Hospital affiliated to Shandong University, Jinan, Shandong, China; 3 Department of Oncology, Linyi Cancer Hospital, Linyi, Shandong, China; 4 Department of Oncology, Jining Infectious Diseases Hospital, Jining, Shandong, China; 5 Department of Radiology, Shandong Cancer Hospital affiliated to Shandong University, Jinan, Shandong, China; 6 Shandong Academy of Medical Sciences, Jinan, Shandong, China; Technische Universitat Munchen, GERMANY

## Abstract

To reduce the high risk of radiation toxicity and enhance the quality of life of patients with non-small cell lung cancer (NSCLC), we quantified the metabolic tumor volumes (MTVs) from baseline to the late-course of radiotherapy (RT) by fluorodeoxyglucose positron emission tomography computerized tomography (FDG PET-CT) and discussed the potential benefit of late-course adaptive plans rather than original plans by dose volume histogram (DVH) comparisons. Seventeen patients with stage II-III NSCLC who were treated with definitive conventionally fractionated RT were eligible for this prospective study. FDG PET-CT scans were acquired within 1 week before RT (pre-RT) and at approximately two-thirds of the total dose during-RT (approximately 40 Gy). MTVs were taken as gross tumor volumes (GTVs) that included the primary tumor and any involved hilar or mediastinal lymph nodes. An original plan based on the baseline MTVs and adaptive plans based on observations during-RT MTVs were generated for each patient. The DVHs for lung, heart, esophagus and spinal cord were compared between the original plans and composite plans at 66 Gy. At the time of approximately 40 Gy during-RT, MTVs were significantly reduced in patients with NSCLC (pre-RT 136.2±82.3 ml vs. during-RT 64.7±68.0 ml, p = 0.001). The composite plan of the original plan at 40 Gy plus the adaptive plan at 26 Gy resulted in better DVHs for all the organs at risk that were evaluated compared to the original plan at 66 Gy (p<0.05), including V_5_, V_10_, V_15_, V_20_, V_25_, V_30_ and the mean dose of total lung, V_10_, V_20_, V_30_, V_40_, V_50_, V_60_ and the mean dose of heart, V_35_, V_40_, V_50_, V_55,_ V_60_, the maximum dose and mean dose of the esophagus, and the maximum dose of the spinal-cord. PET-MTVs were reduced significantly at the time of approximately 40 Gy during-RT. Late course adaptive radiotherapy may be an effective way to reduce the dose volume to the organs at risk, thus reducing radiation toxicity in patients with NSCLC.

## Introduction

Lung cancer is the leading cause of cancer death[1]_._ Approximately 80% to 85% of lung cancer cases are non-small cell lung cancer (NSCLC). Of these cases, over 60% of patients require radiation therapy (RT) during the course of the disease[2]. In recent years, despite modern radiotherapy technologies, such as three-dimensional conformal radiation therapy (3D-CRT) and/or intensity modulation radiation therapy (IMRT), one treatment of 2 Gy is given daily 5 days per week for a total of 60+ Gy over 6+ weeks. The 5-year survival rate of NSCLC is approximately 15–20%[3]. Recent studies have shown that the incidence of severe radiation-induced lung toxicity (RILT) (Grade ≥ 3) is approximately 2.2–18%, and the incidence of radioactive esophagitis (Grade ≥ 3) is approximately 12.5%-34%[4,5,6]. This may be due to unknown changes in the tumor and normal tissues biological characteristics and function during therapy. Recent studies have demonstrated that, compared with prior to radiotherapy, the tumor size, shape, position, biological activity and normal tissue function may change and even cause tumor target and important organ damage in the middle of RT. Therefore, it is necessary to make comprehensive assessments during RT.

Dose escalation has been shown to be an effective means of increasing local control. Every 1 Gy above the conventional prescription dose could improve the 3- to 5-year survival rate by approximately 1% and decrease the hazard of death by 3%[7]. However, the potential high risk of radiation toxicity limits dose escalation[4,5,8,9], and, thus, 3D-CRT and IMRT based on computerized tomography (CT) imaging could not improve the response significantly. Molecular imaging may be the best tool to obtain the dynamic and spatial biological features safely. In recent years, fluorodeoxyglucose positron emission tomography (FDG-PET) has been widely used in patients with NSCLC, such as for the diagnosis and staging of tumors, choice of treatment plan, assessment of treatment responses and delineation of radiotherapy tumor volumes. FDG-PET has enabled image-guided RT to assess the patient’s position and biological characteristics for each treatment[10,11,12,13,14,15,16,17]. While most studies have focused on imaging prior to or after RT, they have ignored tumor changes during RT. Adaptive RT, whereby the treatment plans are modified over the course of treatment to account for individual patient and tumor changes, is an emerging concept for improving local control while minimizing toxicity[18]. It would be preferable to acquire FDG-PET before and during treatment, which could provide an opportunity to adjust and optimize further therapy.

A small pilot study by Kong et al.[19] demonstrated that tumor activity was reduced during-RT (40–50 Gy), and during-RT, PET-metabolic tumor volumes (PET-MTVs) can be used to adapt the radiotherapy plans to provide radiation dose escalation (30102 Gy, mean 58 Gy) to more active malignancies. Meanwhile, it may reduce the normal tissue complication probability (NTCP) by 0.4–3% (mean, 2%)[20]. Stanford University (RSNA2008) has reported that at approximately two-thirds of the total dose during-RT (approximately 4 weeks), the FDG uptake was decreased and associated with a progression free survival rate.

Thus, the purpose of our study was to quantify the change in MTVs from baseline to during-RT and then explore the potential benefits of late-course adaptive plans compared to original plans by dose volume histogram (DVH) comparisons. If the changes can be predicted during treatment, the remaining treatment may be individualized accordingly.

## Materials and Methods

### Ethical approval

The study was approved by the Ethical Committee of the Shandong Cancer Hospital affiliated to Shandong University and has, therefore, been performed in accordance with the ethical standards established in the 1964 Declaration of Helsinki. All patients provided written informed consent before the study.

### Patients

Adult patients with histologically confirmed stage II to III NSCLC (American Joint Committee on Cancer 2003) requiring definitive irradiation with chemotherapy based on the stage of the disease and medical condition were enrolled in this prospective study from March 2013 to October 2014 in the Shandong Cancer Hospital affiliated to Shandong University. Written informed consent was obtained from the subjects before enrollment. Patients with small cell lung cancer or mixed small cell/non-small cell tumor histology, who received pericardial effusion or were pregnant or lactating were excluded from the study.

### ^18^F-FDG PET/CT image

FDG PET-CT images were acquired as previously described[21]. In this study, PET-CT scans were acquired within 1 week before RT (pre-RT) and during RT (during-RT) of approximately 40 Gy. PET-CT scans were evaluated qualitatively by both a nuclear medicine physician and a radiation oncologist and an objective quantification of FDG uptake in the region of interest (ROI).

### PET-MTVs delineation

**Fig 1:**
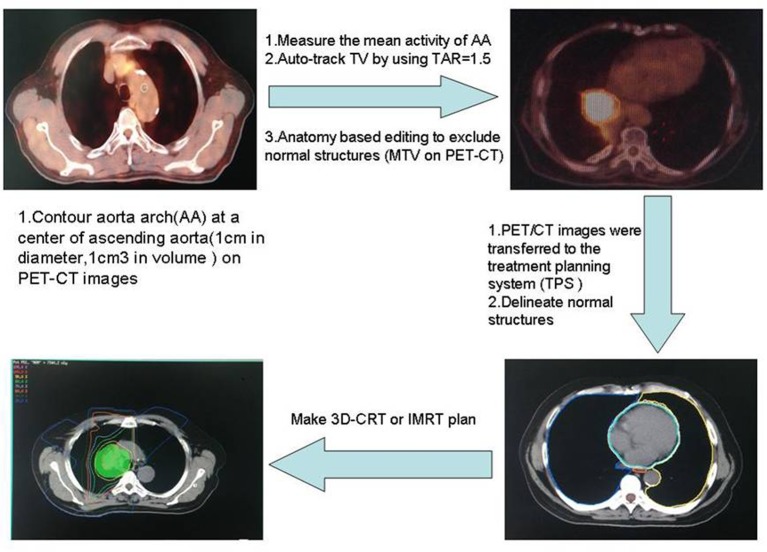
PET-MTVs delineation and treatment planning. PET-MTVs, positron emission tomography-metabolic tumor volumes.

There are multiple ways to define PET-MTVs. We elected to use an auto-segmentation method based on a fixed source/background ratio combined with CT anatomy based manual editing to delineate PET-MTVs, as illustrated in Fig 1. The specific delineation method can refer to what has been described in a previous study[22].

### Treatment planning

PET/CT images were transferred to the treatment planning system (TPS, Varian Medical Systems, Palo Alto, CA, USA) via DICOM. The initial planning target volume (pre-PTV) was based on PET1MTVs. MTVs that were considered the gross tumor volume (pre-GTV) included the primary tumor and any involved hilar or mediastinal lymph nodes. Uninvolved lymph node regions were not included in the clinical target volume (CTV). The initial CTV (pre-CTV) consisted of the pre-GTV and approximately a 0.5 cm margin for microscopic extension. The pre-PTV consisted of the pre-CTV plus a minimal 0.5 cm margin for setup error plus an individualized margin for target motion if the motion was not controlled or the pre-GTV did not include the target motion. The same steps were performed on the first images of the treatment planning and the images taken after 40 Gy. The pre-PTV for the adaptive plan was the during-PTV, which was defined based on the during-RT PET/CT study and consisted of the PET2MTVs plus at least a 0.5 cm expansion. The PET2MTVs were auto-contoured using the same normalized threshold value (normalized to the mediastinal blood pool in the aortic arch) as was used to define the PET1MTVs, pre-GTV, pre-CTV and pre-PTV using the same motion management technique employed in the initial plan.

Radiation was delivered using a 3D-CRT or IMRT to match the planning constraints (mean lung dose to <20 Gy and total lung V20 ≤35%, maximal spinal cord dose ≤45 Gy). An original plan, based on the baseline MTVs, and an adaptive plan, based on during-RT MTVs, were generated for each patient. Both phases of treatment were implemented by 2.0 Gy/fraction, 5 fractions a week. The dose was prescribed to the 90% isodose line with lung correction for inhomogeneity, which encompassed at least 95% of the PTV. The DVHs for lung, heart, esophagus and spinal cord were compared between the original plan at the prescribed dose 66 Gy and the composite plan at 66 Gy (original plan at 40 Gy plus the adaptive plan at 26 Gy).

The PET images were used only to provide an assessment of the metabolic tumor volume, and they were not incorporated quantitatively into the clinical treatment planning. All patients’ prescribed treatment courses were carried out according to the original plan.

### Statistical considerations

The primary objective of this study was to compare the changes in PET-MTVs between pre- and during-treatment and discuss the potential benefits of a late-course adaptive plan compared to the original plan by DVH comparison. SPSS 17.0 software was used to test statistical significance. The change in each individual PET-MTV during RT was compared to that of baseline and the different DVH for all the organs at risk (lung, heart, esophagus and spinal cord) between the original plan at the prescribed dose 66 Gy and the composite plan at 66 Gy (original plan at 40 Gy plus the adaptive plan at 26 Gy). Comparisons were assessed by a nonparametric test (Wilcoxon signed-rank test) or 2 tailed paired t-test. P-values equal to or less than 0.05 were considered statistically significant. Unless otherwise specified, the data are presented as the mean (95% CI).

## Results

### Patient characteristics and treatment

Seventeen stage II to III NSCLC patients were enrolled in this study. Fourteen patients were male, and 3 patients were female. The median age was 64 years. Five patients had Stage II, and 12 patients had Stage III disease (characteristics are shown in Table 1). All patients had FDG PET/CT scans within 1 week prior to the initiation of radiation therapy and after the delivery of approximately 40 Gy (during-RT).

**Table 1:** Patient Characteristics.

Characteristics	Numbers of patients
**Age, years**	
≤70	14
>70	3
**Gender**	
Male	14
Female	3
**KPS**	
≤80	3
>80	14
**Smoker**	
Yes	15
No	2
**Type of cancer**	
Central	10
Peripheral	7
**Histology**	
Adenocarcinoma	3
Squamous carcinoma	10
Not otherwise specified	4
**Clinical stage**	
Ⅱ	5
Ⅲ	12
**Chemotherapy**	
Yes	17
No	0

KPS, Karnofsky.

### Changes in PET-MTVs during RT

**Fig 2:**
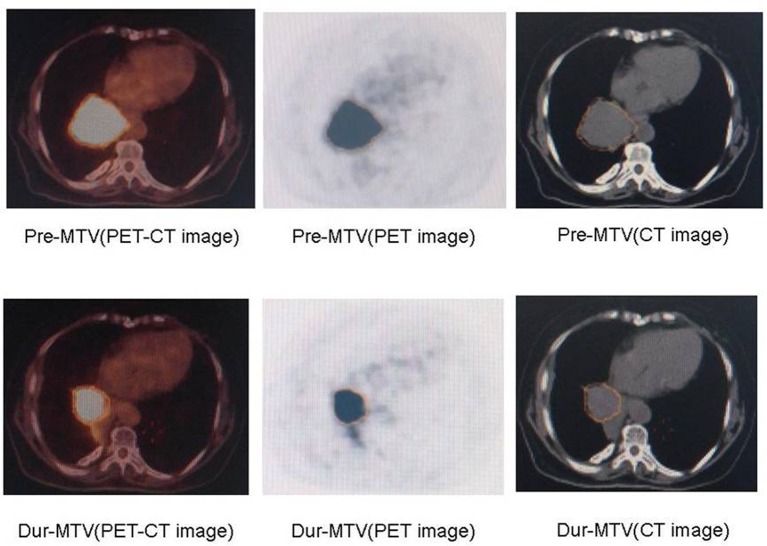
Comparison of MTVs pre-RT and dur-RT. MTVs, metabolic tumor volumes; RT, radiotherapy.

The changes in PET-MTVs during RT are presented in Table 2. Examples of patients with MTV reductions are presented in Fig 2. For the entire group of seventeen patients, compared to the baseline values, the volume of PET-MTVs were reduced significantly at the time of approximately 40 Gy (pre-RT 136.2±82.3 ml vs. during-RT 64.7±68.0 ml, p = 0.001).

**Table 2:** MTV and parameters of DVH for all the organs at risk.

Organs	Parameters	Original plan	Composite plan	p value
**MTV**	(ml)	136.2±82.3*	64.7±68.0*	0.001
**Total-lung**	V5	58.90%	48.60%	<0.001
	V10	44.50%	34.60%	<0.001
	V13	37.40%	30.50%	<0.001
	V15	33.90%	27.80%	<0.001
	V20	27.80%	23.30%	<0.001
	V25	23.70%	19.20%	<0.001
	V30	20.40%	15.60%	<0.001
	D_mean_ (cGy)	1663.0±568.9*	1444.6±547.4*	<0.001
**Heart**	V10	50.20%	38.20%	0.002
	V20	34.60%	28.10%	0.002
	V30	25.60%	21.90%	0.001
	V40	18.20%	15.10%	0.001
	V50	13.30%	9.50%	0.001
	V60	6.50%	4.40%	0.001
	D_mean_ (cGy)	1937.4	1545.8	0.001
**Esophagus**	V35	39.40%	35.70%	0.001
	V40	38.30%	32.50%	0.001
	V50	31.30%	25.90%	0.001
	V55	29.50%	21.60%	0.001
	V60	26.90%	17.80%	0.001
	D_max_ (cGy)	7241.2	7186.9	0.001
	D_mean_ (cGy)	2528.2±1136.3*	2268.3±1196*	0.04
**Spinal-cord**	D_max_ (cGy)	4,199.7	4,133.9	0.004

*: Mean ± standard deviation.

MTV, metabolic tumor volumes; DVH, dose volume histogram.

### Different DVHs between the original plan and composite plan

For all the patients, we completed RT planning before and during radiotherapy. The composite plan of the original plan at 40 Gy plus the adaptive plan at 26 Gy resulted in better DVH for all the organs at risk than the original plan at 66 Gy (p<0.05), including V5 (48.6% vs. 58.9% p<0.001), V10 (34.6% vs. 44.5% p<0.001), V13 (30.5% vs. 37.4% p<0.001), V15 (27.8% vs. 33.9% p<0.001), V20 (23.3% vs. 27.8% p<0.001), V25 (19.2% vs. 23.7% p<0.001), V30 (15.6% vs. 20.4% p<0.001) and the mean dose for the total lung (1444.6±547.4 cGy vs. 1663.0±568.9 cGy p<0.001). V10 (38.2% vs. 50.2% p = 0.002), V20 (28.1% vs. 34.6% p = 0.002), V30 (21.9% vs. 25.6% p = 0.001), V40 (15.1% vs. 18.2% p = 0.001), V50 (9.5% vs. 13.3% p = 0.001), V60 (4.4% vs. 6.5% p = 0.001) and the mean dose for the heart (1545.8 cGy vs. 1937.4 cGy p = 0.001). V35 (35.7% vs. 39.4% p = 0.001), V40 (32.5% vs. 38.3% p = 0.001), V50 (25.9% vs. 31.3% p = 0.001), V55 (21.6% vs. 29.5% p = 0.001), V60 (17.8% vs. 26.9% p = 0.001), the maximum dose (7186.9 cGy vs. 7,241.2 cGy p = 0.001) and the mean dose for the esophagus (2268.3±1196 cGy vs. 2528.2±1136.3 cGy p = 0.04), and the maximum dose for the spinal-cord (4133.9 cGy vs. 4199.7 cGy p = 0.004). The compared parameters collected from DVH for the original plan and the composite plan are listed in Table 2.

## Discussion

Radiotherapy is the main local treatment for patients with inoperable stage II-III NSCLC, and an adequate dose is essential for treatment success as increased radiation doses have been associated with reduction in the risk of death[7]. However, most patients with stage III NSCLC cannot receive an adequate dose for tumor control without exceeding the “safe” dose limits of the adjacent critical structures, such as the lung, esophagus, heart and spinal cord. An accurate definition of a target is crucial for the delivery of high-precision radiotherapy in NSCLC. We used FDG-PET to assess the tumor and normal tissues biological characteristics and function during RT. Meanwhile, FDG-PET could play an important role in lymph node staging by accurately showing positive nodes. However, PET helped to differentiate tumors and collapsed lungs, allowing a more accurate radiation volume of the lung. To redirect the remaining treatment, FDG PET-CT scans were repeated at approximately two-thirds of the total dose during-RT (approximately 40 Gy).

This study demonstrated that PET-MTVs may be reduced significantly at the time of approximately 40 Gy during RT (pre-RT 136.2±82.3 ml vs. during-RT 64.7±68.0 ml, p = 0.001), and late-course adaptive radiotherapy may be an effective way to reduce the dose volume to the organs at risk in patients with NSCLC, which deserves further study. In a previous study, Mahasittiwat et al.[22] found that the mean PET-MTVs were 43.4 cc (28.2–58.5 cc) and 17.9 cc (10.0–25.7 cc) on pre-RT and during-RT (approximately 45 Gy) scans, respectively, and the mean reduction in PET-MTVs was 32.2 cc (20.8–43.7 cc, p<0.001). In the course of RT (40 Gy~50 Gy), FDG-PET/CT activity decreased[20], and the tumor volume was significantly reduced[7,23,24,25,26]. Similar results have been obtained in previous studies. A study by Fox et al.[27] indicated that the GTV had a median reduction during RT (24.7% at 30 Gy, 44.3% at 50 Gy). Ding et al.[25] carried out an analogous study that demonstrated that the tumor volumes regressed in 84 (96.6%) patients and increased in 3 (3.4%) patients after 40 Gy; the mean GTV and PTV reduction was 38% (range, 213–95%) and 30% (range, 25–95%). Guckenberger et al.[28] studied the tumor changes in 13 patients with NSCLC, and they found continuous tumor regression by 1.2% per day as measured by CT images, which was in good agreement with the results presented by Kupelian’s research[29] and Everitt’s research[26].

PET-MTVs were significantly reduced in RT, such that it may directly lead to improvements in lung function, toxicity rates and dose escalation. While we still do not know if the target volume reductions are warranted when GTV shrinkage has occurred during RT[29,30,31], some physicians advocated an approach in which the target volume remained constant due to concerns of residual microscopic disease. In our study, the composite plan resulted in better DVH for all the organs at risk that were evaluated compared to the original plan at 66 Gy (p<0.05), including V_5_, V_10_, V_15_, V_20_, V_25_, V_30_ and the mean dose (p<0.05) for the total lung; V_10_, V_20_, V_30_, V_40_, V_50_, V_60_ and the mean dose (p<0.05) for the heart; V_35_, V_40_, V_50_, V_55_, V_60_, the maximum dose and mean dose (p<0.05) for the esophagus; and the maximum dose (p<0.05) for the spinal-cord. Ding et al.[21] found that during RT (40 Gy), after modifying the RT plan, the dose volume parameters of V20 of the lung, V60 of the esophagus, V45 of the heart, V65 of the heart in the composite plan (40 Gy to the PTV with a subsequent 20–28 Gy-boost to the shrunken PTV) were higher than original plan (delivering the same dose to the initial PTV without shrinking the field; p<0.05). Feng et al.[20] performed a pilot study in which 14 patients with NSCLC underwent PET-CT scans pre-RT and during RT. They found that this method allowed for a mean dose escalation of 58 Gy or a reduction in NTCP of up to 3% in patients with a reduction in tumor size. Similar results could be seen in another study by Gillham et al.[32].

To the best of our knowledge, we aimed to examine the MTVs from baseline to the late-course of RT by FDG PET-CT and discuss the potential benefits of late-course adaptive plans compared to the original plans by DVH comparisons. There are several limitations to this study. (1) As the sample size was small, further study with a larger number of patients is needed to validate the present findings. (2) This was only a planning study, we do not know if this adaptive plan affected tumor control or increased the rate of tumor recurrence with the narrowed treatment fields. All these limitations require further evaluation in future studies.

## Conclusion

This study indicated that PET-MTVs may be reduced significantly at the time of approximately 40 Gy during RT, and late course adaptive radiotherapy may be an effective way to reduce the dose volume to the organs at risk. This could reduce radiation-induced injury in patients with NSCLC. More prospective studies on FDG PET-CT scans are ongoing in our institution.

## Supporting Information

S1 Table MTVs of ^18^F-FDG PET-CT in original plans and composite plans. (XLS)

S2 Table Parameters of DVH for all the organs at risk in original plans and composite plans. (XLS)

## References

[1] Parkin DM, Bray F, Ferlay J, Pisani P. Global cancer statistics, 2002. CA Cancer J Clin 2005;55:74–108. 15761078 10.3322/canjclin.55.2.74

[2] Tyldesley S, Boyd C, Schulze K, Walker H, Mackillop WJ. Estimating the need for radiotherapy for lung cancer: an evidence-based, epidemiologic approach. Int J Radiat Oncol Biol Phys 2001;49:973–85. 11240238 10.1016/s0360-3016(00)01401-2

[3] Aupérin A, Le PC, Rolland E, Curran WJ, Furuse K, et al (2010) Meta-analysis of concomitant versus sequential radiochemotherapy in locally advanced non-small-cell lung cancer. J Clin Oncol 28: 2181–2190. 10.1200/JCO.2009.26.2543 20351327

[4] Barriger RB, Fakiris AJ, Hanna N, Yu M, Mantravadi P, McGarry RC. Dose-volume analysis of radiation pneumonitis in non-small-cell lung cancer patients treated with concurrent cisplatinum and etoposide with or without consolidation docetaxel. Int J Radiat Oncol Biol Phys 2010;78:1381–6. 10.1016/j.ijrobp.2009.09.030 20231061

[5] Werner-Wasik M, Paulus R, Curran WJ, Byhardt R. Acute esophagitis and late lung toxicity in concurrent chemoradiotherapy trials in patients with locally advanced non-small-cell lung cancer: analysis of the radiation therapy oncology group (RTOG) database. Clin Lung Cancer 2011;12:245–51. 10.1016/j.cllc.2011.03.026 21726824

[6] Flentje M, Huber RM, Engel-Riedel W, Andreas S, Kollmeier J, et al (2016) GILT—A randomised phase III study of oral vinorelbine and cisplatin with concomitant radiotherapy followed by either consolidation therapy with oral vinorelbine and cisplatin or best supportive care alone in stage III non-small cell lung cancer. Strahlenther Onkol 192: 216–222. 10.1007/s00066-016-0941-8 26809652

[7] Kong FM, Ten HRK, Schipper MJ, Sullivan MA, Chen M, Lopez C, et al High-dose radiation improved local tumor control and overall survival in patients with inoperable/unresectable non-small-cell lung cancer: long-term results of a radiation dose escalation study. Int J Radiat Oncol Biol Phys 2005;63:324–33. 10.1016/j.ijrobp.2005.02.010 16168827

[8] Phernambucq EC, Spoelstra FO, Verbakel WF, Postmus PE, Melissant CF, van den Brink KI M, et al Outcomes of concurrent chemoradiotherapy in patients with stage III non-small-cell lung cancer and significant comorbidity. Ann Oncol 2011;22:132–8. 10.1093/annonc/mdq316 20595452

[9] Saitoh J, Saito Y, Kazumoto T, Kudo S, Yoshida D, Ichikawa A, et al Concurrent chemoradiotherapy followed by consolidation chemotherapy with bi-weekly docetaxel and carboplatin for stage III unresectable, non-small-cell lung cancer: clinical application of a protocol used in a previous phase II study. Int J Radiat Oncol Biol Phys 2012;82:1791–6. 10.1016/j.ijrobp.2011.03.007 21601375

[10] Juweid ME, Cheson BD. Positron-emission tomography and assessment of cancer therapy. N Engl J Med 2006;354:496–507. 10.1056/NEJMra050276 16452561

[11] Ford EC, Herman J, Yorke E, Wahl RL. 18F-FDG PET/CT for image-guided and intensity-modulated radiotherapy. J Nucl Med 2009;50:1655–65. 10.2967/jnumed.108.055780 19759099 PMC2899678

[12] Lee P, Kupelian P, Czernin J, Ghosh P. Current concepts in F18 FDG PET/CT-based radiation therapy planning for lung cancer. Front Oncol 2012;2:71 10.3389/fonc.2012.00071 22798989 PMC3393879

[13] Zheng Y, Sun X, Wang J, Zhang L, DI X, Xu Y. FDG-PET/CT imaging for tumor staging and definition of tumor volumes in radiation treatment planning in non-small cell lung cancer. Oncol Lett 2014;7:1015–20. 10.3892/ol.2014.1874 24944661 PMC3961455

[14] Spratt DE, Diaz R, McElmurray J, Csiki I, Duggan D, Lu B, et al Impact of FDG PET/CT on delineation of the gross tumor volume for radiation planning in non-small-cell lung cancer. Clin Nucl Med 2010;35:237–43. 10.1097/RLU.0b013e3181d18eb0 20305410

[15] Mac MMP, Hicks RJ. The role of positron emission tomography/computed tomography in radiation therapy planning for patients with lung cancer. Semin Nucl Med 2012;42:308–19. 10.1053/j.semnuclmed.2012.04.003 22840596

[16] Chi A, Nguyen NP. The utility of positron emission tomography in the treatment planning of image-guided radiotherapy for non-small cell lung cancer. Front Oncol 2014;4:273 10.3389/fonc.2014.00273 25340040 PMC4187610

[17] Marta GN, Hanna SA, de Camargo Etchebehere EC S, Camargo EE, Haddad CM, Fernandes dSJL. The value of positron-emission tomography/computed tomography for radiotherapy treatment planning: a single institutional series. Nucl Med Commun 2011;32:903–7. 10.1097/MNM.0b013e32834a719a 21876401

[18] Lim G, Bezjak A, Higgins J, Moseley D, Hope AJ, et al (2011) Tumor regression and positional changes in non-small cell lung cancer during radical radiotherapy. J Thorac Oncol 6: 531–536. 10.1097/JTO.0b013e31820b8a52 21258244

[19] Kong FM, Frey KA, Quint LE, Ten HRK, Hayman JA, Kessler M, et al A pilot study of [18F]fluorodeoxyglucose positron emission tomography scans during and after radiation-based therapy in patients with non small-cell lung cancer. J Clin Oncol 2007;25:3116–23. 10.1200/JCO.2006.10.3747 17634490

[20] Feng M, Kong FM, Gross M, Fernando S, Hayman JA, Ten HRK. Using fluorodeoxyglucose positron emission tomography to assess tumor volume during radiotherapy for non-small-cell lung cancer and its potential impact on adaptive dose escalation and normal tissue sparing. Int J Radiat Oncol Biol Phys 2009;73:1228–34. 10.1016/j.ijrobp.2008.10.054 19251094 PMC3381895

[21] Ding XP, Zhang J, Li BS, Li HS, Wang ZT, Yi Y, et al Feasibility of shrinking field radiation therapy through 18F-FDG PET/CT after 40 Gy for stage III non-small cell lung cancers. Asian Pac J Cancer Prev 2012;13:319–23. 22502693 10.7314/apjcp.2012.13.1.319

[22] Mahasittiwat P, Yuan S, Xie C, Ritter T, Cao Y, Ten HRK, et al Metabolic Tumor Volume on PET Reduced More than Gross Tumor Volume on CT during Radiotherapy in Patients with Non-Small Cell Lung Cancer Treated with 3DCRT or SBRT. J Radiat Oncol 2013;2:191–202. 10.1007/s13566-013-0091-x 23795245 PMC3686305

[23] Yuan ST, Frey KA, Gross MD, Hayman JA, Arenberg D, Cai XW, et al Changes in global function and regional ventilation and perfusion on SPECT during the course of radiotherapy in patients with non-small-cell lung cancer. Int J Radiat Oncol Biol Phys 2012;82:e631–8. 10.1016/j.ijrobp.2011.07.044 22197235 PMC3381888

[24] Meng X, Frey K, Matuszak M, Paul S, Ten HR, Yu J, et al Changes in functional lung regions during the course of radiation therapy and their potential impact on lung dosimetry for non-small cell lung cancer. Int J Radiat Oncol Biol Phys 2014;89:145–51. 10.1016/j.ijrobp.2014.01.044 24725697 PMC4400732

[25] Ding X, Li H, Wang Z, Huang W, Li B, Zang R, et al A clinical study of shrinking field radiation therapy based on (18)F-FDG PET/CT for stage III non-small cell lung cancer. Technol Cancer Res Treat 2013;12:251–7. 10.7785/tcrt.2012.500310 23289475

[26] Everitt SJ, Ball DL, Hicks RJ, Callahan J, Plumridge N, Collins M, et al Differential (18)F-FDG and (18)F-FLT Uptake on Serial PET/CT Imaging Before and During Definitive Chemoradiation for Non-Small Cell Lung Cancer. J Nucl Med 2014;55:1069–74. 10.2967/jnumed.113.131631 24833494

[27] Fox J, Ford E, Redmond K, Zhou J, Wong J, Song DY. Quantification of tumor volume changes during radiotherapy for non-small-cell lung cancer. Int J Radiat Oncol Biol Phys 2009;74:341–8. 10.1016/j.ijrobp.2008.07.063 19038504

[28] Guckenberger M, Wilbert J, Richter A, Baier K, Flentje M. Potential of adaptive radiotherapy to escalate the radiation dose in combined radiochemotherapy for locally advanced non-small cell lung cancer. Int J Radiat Oncol Biol Phys 2011;79:901–8. 10.1016/j.ijrobp.2010.04.050 20708850

[29] Kupelian PA, Ramsey C, Meeks SL, Willoughby TR, Forbes A, Wagner TH, et al Serial megavoltage CT imaging during external beam radiotherapy for non-small-cell lung cancer: observations on tumor regression during treatment. Int J Radiat Oncol Biol Phys 2005;63:1024–8. 10.1016/j.ijrobp.2005.04.046 16005575

[30] Ramsey CR, Langen KM, Kupelian PA, Scaperoth DD, Meeks SL, Mahan SL, et al A technique for adaptive image-guided helical tomotherapy for lung cancer. Int J Radiat Oncol Biol Phys 2006;64:1237–44. 10.1016/j.ijrobp.2005.11.012 16446055

[31] Siker ML, Tomé WA, Mehta MP. Tumor volume changes on serial imaging with megavoltage CT for non-small-cell lung cancer during intensity-modulated radiotherapy: how reliable, consistent, and meaningful is the effect. Int J Radiat Oncol Biol Phys 2006;66:135–41. 10.1016/j.ijrobp.2006.03.064 16839704

[32] Gillham C, Zips D, Pönisch F, Evers C, Enghardt W, Abolmaali N, et al Additional PET/CT in week 5–6 of radiotherapy for patients with stage III non-small cell lung cancer as a means of dose escalation planning. Radiother Oncol 2008;88:335–41. 10.1016/j.radonc.2008.05.004 18514339

